# High serum estradiol confers no risk for breast cancer: another disparity for sub Saharan Africa women

**Published:** 2012-06-06

**Authors:** John Peter Awio, Moses Galukande, Olivia Kituuka, Jane Odubu Fualal

**Affiliations:** 1 Department of Surgery, School of Medicine, College of Health Sciences, Makerere University, Uganda

**Keywords:** Oestradiol, risk, breast cancer, women, sub-sahara Africa

## Abstract

**Introduction:**

There are breast cancer epidemiological and tumor behaviour disparities between black women in sub Saharan Africa and their counter parts in western high resource countries. In Uganda, the incidence of breast cancer has nearly tripled in over a four decades for uncertain reasons. High serum estradiol is a known risk factor for breast cancer among women in high resourced nations. The objective of this study was to establish whether high serum estradiol is an associated risk for breast cancer amongst a group of black Ugandan women.

**Methods:**

A case control study, conducted over eight month period with incident breast cancer as cases and the controls were without breast cancer but at risk and representative of the population from which the cases were chosen. Questionnaires were administered, clinical examination was done, serum estradiol level estimation was done using cobase immunoassay analyzer using Electro chemiluminescence Immuno assay (ECLIA). Data was analyzed using logistic regression model, and a p - value of less than 0.05 was considered significant. IRB approval was secured.

**Results:**

A total of 140 women participated, 70 cases and 70 controls. The median estrogen levels was 43.2 pg/ml with IQR of 18.48 to 75.8 pg/ml, the value was higher among premenopausal women than those without cancer but with no statistical significance. No association was found between level of estradiol and breast cancer (p 0.647). The median oestrogen levels were significantly higher than normal levels in Caucasian women.

**Conclusion:**

There was no association between level of estradiol and breast cancer. This is yet another disparity between women of African origin and the non Africans in high resourced countries. There is need to explore more to explain this disparity.

## Introduction

Breast cancer is the most common type of cancer diagnosed in women world wide, excluding skin cancer. Globally almost one third (32%) of all cancers diagnosed in women are breast cancer [1]. In Uganda breast cancer is the third commonest cancer in women after cancer of the cervix and Kaposi′s sarcoma [2]. The incidence of breast cancer in Uganda has tripled from 11:100,000 in 1961 to 31:100,000 in 2006 [3, 4], the tumors are seen present in relatively young women, mostly late in stage III and IV, run an aggressive course and carry a low 5 year survival rate [5].Whereas in the western world the incidence of breast cancer and many other cancers is stabilizing and even on a decline, in Uganda and other sub-Saharan countries it's on the increase [4, 6], the reasons for those disparities are yet to be fully elucidated.

High oestradiol exposure (≥ 8.03pg/ml) has been found to be a strong indicator for development of breast cancer, women with high levels have an up to 3.6 fold greater risk for breast cancer compared to women within the low levels [7].

The purpose of this study therefore was to explore associations between high estradiol level and breast cancer among Ugandan women, as part of the mission to understand the apparent disparities between women in low resourced sub-Saharan Africa and the western high resourced nations. Ultimately the understanding of underlying biological mechanisms may contribute to novel therapeutic interventions.

## Methods

### Study design

A case control study.

### Study setting

Mulago hospital is a national referral and teaching hospital for Makerere University in central Uganda 2km from the city center. It has a bed capacity of 1500. The study was done at the Breast unit. It is the only public institution with comprehensive specialist-based and free breast cancer screening and care facilities. The unit receives at least three cases of incident breast cancer and five patients for the screening per week; an outpatient clinic runs once a week, the other services, radiotherapy, surgery, chemotherapy run through out the week. Primary care physicians recognize this center as the only free medical facility with comprehensive breast cancer specialists.

### Study population

Women who came to the breast unit with suspected breast cancer or those screened for breast cancer at Mulago Hospital during the six months study period who met the eligibility criteria and given an informed consent to participate in the study.

### Procedure

All women presenting with incident breast cancer, confirmed by histology who were able to give relevant information for the study and consent to participate in the study were the cases. Women without breast diseases and had normal mammographic findings, who were able to give relevant information and consent for the study were the controls selected from patients that presented for screening and breast health care. All women with benign breast lesions, severely ill or unable to give relevant information, or pregnant, on HRT, contraceptive pills or diagnosed with any form of cancer were excluded. In the clinic patients were screened, history and physical examinations were done to exclude other conditions. Those without breast lump but within the age group for breast cancer screening were sent for mammography and are reviewed with mammography results. Those with lesion detected by mammography, went for Fine Needle Aspiration Cytology (FNAC). Those with clinically palpable masses were sent for mammography and FNAC. Further management followed depending on the results of the test. Needle and excision biopsies were the main procedures done in the clinic. When participants had consented and had gone through examination and investigations. They were divided into 2 groups, Women with a histological diagnosis of breast cancer were recruited in the study as cases while those with normal breast, were recruited in the study as controls. All information was recorded in a data collection form. The data collection method was an interviewer-administered questionnaire. The patients were recruited from the breast clinic.

Serum specimens were obtained from all participants who adhered to a fat-free diet during the night and morning before the sample collection to minimize lipemia that could interfere with assays. Blood was drawn (phlebotomy), after the participant had consented. 3ml of venous blood was taken in a vacuum container with coagulant. For pre-menopausal women blood was taken only within the first seven days from the first day of menstrual flow since this period the estrogen level is not under the influence of physiology of menstruation.

Serum was immediately analyzed or frozen, at negative 20°C. The sample was safe at this temperature for six months. Assays were performed concurrently on serum specimens from cases and controls. Cobase immunoassay analyzers using Electro chemilumine scence Immuno assay (ECLIA). Reagents used streptauidia coated micro-particles (M), anti estradiotab-biotin 9(R1) and estradiol-peptide-RU (bpy) R2. The lab was blinded to the case status. Reproducibility was determined using elecsys agents. Pooled human sera and controls in a modified protocol in a modified protocol (EP5-A) of the NCCLS (National Committee for clinical lab standards).

The questionnaire was pre tested to check whether it extracts the desired information. Data was checked for completeness before the participants are discharged from the study. Analysis was done using SPSS version 16 logistical regression model used, p-value Sample size calculation for estimation of association of serum estradiol with breast cancer; we determined it by Fleiss method with the correction factor [8] that 70 women per group would be necessary to detect an odd ratio of 2.5; with 80% power at a significance level of 0.05.

### Ethical considerations

Informed consent was obtained from the participants. Appropriate ethical approval was sought.

## Results

All 140 participants were female; 70 were cases and 70 controls. Pre tested questionnaires were administered, participants were from various parts of the country with majority being the Bantu ethnic group. The average age of the respondents was 45 year in the cases and 44 years in the controls (Table 1).

**Table 1:** Comparison of the study variables in two groups

	Cases (N = 70)	Controls (N = 70)	Group difference 95% CI (mean/proportion)	P value
Age	44.6	43.8	-3.37 to 4.97	0.705
Body mass index	24.5	26.4	-3.46 to -0.50	0.009
Menarche before 12 years	2	11	-0.22 t0 -0.04	0.009
Menopause after 55 years	28	20	-0.04 to 0.27	0.441
Surgical menopause	4	2	-0.04 to 0.10	0.877
Number of women on Contraceptives	31	34	-0.21 to 0.12	0.556
Number of pregnancies (average)	5.4	5.1	-0.92 to 1.33	0.720
Age at first pregnancy	18.9	19.5	-1.76 to 0.61	0.355
Years breastfed	8.2	8.0	-1.45 to 1.97	0.763
Women on HRT	0	3	-0.09 to 0.01	0.08
Family history of breast cancer	8	6	-0.07 to 0.13	0.592
Ever diagnosed breast cancer	0	1		0.316
Ever diagnosed benign breast disease	6	25		0.001
Patients taking alcohol	3	11	-0.21 to 0.02	0.024
Patients who smokes	3	5		0.466

The distribution of factors in both the cases and the controls was mostly the same (Table 1). However significant differences were noted in the body mass index, age of menarche, history of hormonal replacement therapy, history of having been diagnosed with breast cancer and alcohol intake.

Cases had significantly lower body mass index than the controls. The age at menarche was significantly between the cases and controls however the numbers of cases were very small. There were no cases in the ever taken hormonal replacement therapy. Fewer cases than controls had ever been diagnosed with benign breast disease. Alcohol intake was greater in the control group than the cases.

### Serum levels of women with breast cancer and the controls

There was no difference in the median serum estradiol levels in the cases and controls. We could thus say that the median serum estradiol level in the study population was 43.2 pg/ml with interquartile range of 18.48 to 75.8 pg/ml. In this the control group is taken as the normal population representation (Table 2, Table 3).

**Table 2:** Median serum estradiol levels in breast cancer cases and controls

Serum estradiol levels (pg/ml)	Median	25% quartile	75% quartile	Quartile range	P value
Cases	39.7	20.79	87.04	66.25	0.770
Controls	43.2	18.48	75.48	57.00	

**Table 3:** Variation of the estradiol levels by menopausal status

Risk factor	N	Cases (70)	Controls (70)	Mean difference of oestrodial levels	P value
Premenopausal	54	82.18	133.54	51.3	0.474
Postmenopausal	84	58.97	45.48	13	0.6119

No difference was noted in the estradiol levels in the breast cancer cases and their controls in the premenopausal and the post menopausal though the post menopausal women had significantly lower estradiol levels in both the cases and the controls.

### Association of breast cancer risks factor with breast cancer disease

The odds ratio (OR) of developing breast cancer disease increased by 1.02 (95% CI 1.01, 1.04) for each unit increase in body mass index (BMI). Late onset of menarche associated had an Odds Ratio of 0.68 (95% CI 0.52, 0.90) for developing breast cancer compared to women with early menarche, but this was not significant. History of having had hormonal replacement therapy was associated with an OR of 0.60 (95% CI 0.34 – 1.06) of developing breast cancer disease. History of having diagnosed with a benign breast disease is associated with OR of 0.65 (95% CI) for developing breast cancer disease. Alcohol intake was associated with OR of 0.73 (95% CI 0.55 – 0.96) of developing breast cancer (Table 4).

**Table 4:** Association of the risk factors with breast cancer disease risk factor

Risk factor	OR	CI	P value
Age	1.00	0.99,1.01	0.704
BMI	1.02	1.01,1.04	0.008
Age at menarche	0.68	0.52,0.90	0.008
Age at menopause	1.15	0.80,1.64	0.447
Surgical menopause	1.03	0.68,1.57	0.880
Contraceptives	0.95	0.80,1.12	0.558
Number of pregnancies	0.99	0.96,1.02	0.720
Age at first pregnancy	1.01	0.98,1.04	0.354
Years breastfed	1.00	0.97,1.01	0.763
Hormone replacement therapy	0.60	0.34,1.06	0.078
Family history of breast cancer	1.08	0.82,1.42	0.585
Ever diagnosed breast cancer	0.60	0.23,1.62	0.317
Ever diagnosed benign breast disease	0.67	0.56,0.82	0.001
Alcohol intake	0.73	0.55,0.96	0.023
Smokes	0.88	0.61,1.25	0.469

## Discussion

The central role of hormones in the etiology of breast cancer is support by multiple sources of evidence. In several epidemiology studies the role of endogenous hormones in risk of breast cancer among postmenopausal women has been delineated. A strong positive association between breast cancer risk and circulating levels of oestrogen has been well confirmed among postmenopausal women. Evidence among premenopausal women is more limited [9].

In this study we found that high serum estrodial levels conferred no risk to both pre and post menopausal women in this sub Saharan African region but we also found that the serum oestrodiol levels in the control group was higher than that recorded in studies among Caucasian women in general.

### Breast cancer risk factors

Breast cancer is considered to be associated with various documented risk factors. In this study the body mass index, age of menarche, history of ever been diagnosed with benign breast disease and alcohol intake were found to be associated with breast cancer disease.

High BMI was associated with a marginally increase risk of breast cancer, this finding is supported by other previous studies done [10]. This is considered so because the body fat is thought to be the source of estrogen which when continuously exposed to put the women at risk of breast cancer. Late menarche was associated in a marginally protective effect to developing breast cancer. This is similar to previous studies done [11, 12] where age at menarche before 12 years is thought to increase duration of exposure to estrogen leading to breast cancer risk. Having ever been diagnosed with a benign breast mass is also thought to increase the odds of having breast cancer, however in this study it was protective and p-value was significant, the number involved were rather small perhaps introducing possibility of chance occurrence.

### Serum estradiol levels in Ugandan population

Estrogens levels have shown great variations across different continents and ethnic populations world wide [13, 14]. The variation also occurs with the status of the woman being premenopausal or post menopausal and also in the cases and the controls. In our study the median serum estradiol level was 43.2 pg/ml with interquartile range of 18.48 to 75.8 pg/ml. This finding was taken from the control group which is considered to have ‘normal’ estrogen levels. These normal levels are comparatively higher than recorded among Caucasian women. The apparent explanation for comparatively high oestrogen levels is enhanced aromatase activity among black women [15, 16].

### Estradiol levels and the risk of breast cancer disease

High levels of estradiol is associated with an increase in breast cancer risk in different parts of the world with serum estradiol ≥29.4pmol/L having 2.9 to 3.6 relative risk for breast cancer compare to those on the lower quartile ≤18.4pmol/L [7]. In this study done in Uganda, no significant association was found between serum estradiol levels among the studied group of Ugandan women and the risk of breast cancer. This may suggest that high estradiol levels is not a risk factor for breast cancer disease in this study population among both pre and post menopausal women. This is contrary to some of the previous studies done in Europe and America which demonstrated an increasing risk to breast cancer to rising estrogen levels [7]. There could be biological differences in the population studied, underpinning the scope of disparities of breast cancer among black women and therefore the need to explore the real reasons for this disparity perhaps genetics or interplay of genetics and the environment.

### Study limitations

Single samples were collected and analysed once, this may have introduced variations since dynamic changes in estradiol levels occur across a normal menstrual cycle therefore there was additionally limited ability to evaluate associations within specific parts of the cycle. Another factor that may have introduced bias and was not controlled for is the body mass index. Being a hospital based study; both the cases and controls may not be generalizable to the entire country's population, also recall bias may have been an issue perhaps compromising the accuracy of history given. Choice of controls using mammography is affected by the low sensitivity of mammography. It's also difficult to establish a causal relationship since we do not know whether the cause preceded the effect.

## Conclusion

No association was found between level of estradiol and the risk for breast cancer disease among this group of black women in eastern Africa, however the serum oestradiol levels were comparatively high in the normal (controls) compared to studies among Caucasian women, raising the question of enhanced aromatase activity and therefore requiring further studies in this indigenous African context.
